# Disorders of Digestion

**Published:** 1887-01-29

**Authors:** 


### DISORDERS OF DIGESTION.*

(*Continued from p.* 223.)

### THE MOUTH.

"WITH stupidity and sound digestion a man may front much," says Carlyle. Our business just now is with the "sound digestion," not with the "stupidity," though that too is not without its value on occasion. If we had a magic wand, which, with a flourish, would restore the dyspeptic to immediate health, we should soon be the centre of a converging host of pilgrims, who would flock hither from the remotest bounds of the earth. But, for the dyspeptic, as for others, there is no success without effort, no reward without labour. Dr. Lauder Brunton devotes nearly four hundred laborious octavos to the instruction of dyspeptics, and of the doctors who manage them. But even this amount of labour will be ineffectual unless the dyspeptic himself will "gird up the loins of his mind," as St. Paul robustly says, and do his part of the often tedious task. It is rather a melancholy reflection that so many effective brains should be required for the comfort of dyspeptics alone. Indigestion! What a poor thing it is! The robust man regards it with contempt. But he who looks with scorn upon the man who is too conscious of his stomach and liver, must despise civilisation itself. Hitherto, one of the inevitable penalties of progress has been an unhappy digestion. Apparently it must always be so. Not that there is any pre-established necessity for so dire a fate. On the contrary, if all men would be wise there would be no indigestion. But all men will not be wise, any more than they will all wear bottle-green coats, or dye their hair canary-colour, like Tittlebat Titmouse. Dyspepsia is a dreary subject to read and write about, but a still more dreary companion for a permanency; and so, in the interests of those who suffer from it, and with the memory of a "fellow-feeling which makes us wondrous kind," we will, on the lines laid down by Dr. Brunton, essay to give a little instruction and comfort to those who need it.

It is fair to premise that there is no royal road. Dr. Brunton is a man of science, and of all stern mistresses Science is the most hard and stony. She knows absolutely nothing of weak and yielding good-nature. Her "thou shalts" and "thou shalt nots" must be obeyed under fear of the most rigorous penalties. No Moses with the power of stoning; no Elijah with the command of fire from heaven, could visit offenders with direr vengeance than lies in the hands of implacable and unpitying science. But whilst this is so, the converse is equally true, and those who obey with true and steady purpose, seldom fail of their reward.

Like a true man of science, Dr. Brunton begins at the beginning. He tells us first how natural digestion is performed. This, to the doctor, is a very instructive and interesting part of his book. It is doubtful whether it could possibly be made *interesting* to the general reader; but *instructive* it certainly is, and on that account well worthy of perusal.

It is a curious speculation, especially for a certain school of philosophy, why metaphysics, *et hoc genus omne*, should have engaged the attention of mankind at large from the earliest times, whilst physiology has always been regarded, except by a strictly limited few, as a most tiresome and practically unnecessary study. It was apparently the original intention of nature—whatever nature may be—that the body should work out its destiny without any special regard or consideration from its possessor—man. But, in the changed circumstances of modern times, the body refuses to work out its destiny with anything like comfort, and hence we *must* study it whether we will or not. And the results of our researches are clearly these, that we must either revert to a more primitive mode of living, or we must adapt our work and habits intelligently to the capabilities of our physical organism, or—and this is the final alternative—we must in a few decades or centuries work ourselves completely out, and be followed by more vigorous and less civilised races. Let us to our task then, and try so to understand ourselves and our environment as to make provision for continuity and progressive expansion.

Normal digestion is our first study. This presupposes wholesome and suitable food, and *intelligent cookery*. Here is an inviting field for lamentation, diatribe, and sarcasm; but we forbear. Everybody knows where cooks come from. But granted the wholesome food and the intelligent cookery, the process of digestion commences in the mouth; that, at any rate, is no mystery. It is equally clear that good teeth—nature's or the dentist's—are of no small importance. But the food requires more in the mouth than grinding. The buccal cavity is a chemical mixer, as well as a hopper and grinder. There are two or three kinds of saliva poured into it, and a wonderful active principle called Ptyalin, without all of which the mouth can by no means perform its due share in the function of digestion. But if all the saliva is to be good in kind, and sufficient in quantity, there must be healthy blood from which to extract it, and efficient glands to perform the extraction, and regulating nerves to stimulate and control the process, and a vigorous nerve-centre or centres to supply the secretory and controlling fibres with constant and adequate energy, so that before the food leaves the mouth at all, a variety of complex operations must take place, which are more than sufficient to bewilder any learner who is not resolute to master all difficulties, and to gain knowledge at all costs. Whoso wishes to study this initial process in detail, may consult Dr. Brunton's book. We propose to give one or more articles in continuation of the subject in future numbers.

---

* *Disorders of Digestion.* By T. LAUDER BRUNTON, M.D., F.R.S. London: Macmillan and Co.

